# Dynamic Epidural Monitoring of Spinal Cord Neural Conduction Using a Novel Implantable Electrodiagnostic Sensor: A Pre Clinical Study

**DOI:** 10.1177/2689288X251394340

**Published:** 2025-11-11

**Authors:** Babak Shadgan, Alexander Burden, Jocelyn Bégin, Min Lu, Shahbaz Askari

**Affiliations:** ^1^ International Collaboration on Repair Discoveries, Vancouver, Canada.; ^2^ Department of Orthopaedics, University of British Columbia, Vancouver, Canada.; ^3^ School of Biomedical Engineering, University of British Columbia, Vancouver, Canada.

**Keywords:** spinal cord, spinal cord injury, electrodiagnostic monitoring, epidural, electrophysiology, evoked potentials, neurophysiological assessment, rat model

## Abstract

To evaluate the feasibility and diagnostic sensitivity of a novel catheter-based epidural electrodiagnostic (EDX) system for real-time, segment-specific monitoring of spinal somatosensory conduction in a pre-clinical model of acute spinal cord injury (SCI). A custom-designed EDX electrode catheter was epidurally placed over the thoracic spinal cord in anesthetized rats (*n* = 5) to record compound evoked potentials across five stages: Baseline, Hypoxia, Post-SCI, Post-SCI Hypoxia, and Post-SCI Recovery. Waveform morphology, onset/peak latencies, and amplitudes were extracted. Paired *t*-tests compared baseline to experimental stages, and analysis of covariance (ANCOVA) assessed injury force effects on conduction metrics. Postmortem recordings confirmed the biological origin of signals. The EDX system consistently recorded high-fidelity biphasic spinal evoked responses. SCI induced significant increases in N-Onset latency across all post-injury stages (Cohen’s *d* = 2.45–3.04), while N-Peak and P-Peak latencies also increased significantly Post-SCI (Cohen’s *d* = 2.03–2.57), reflecting conduction slowing and partial demyelination. ANCOVA revealed that injury force had large effects on N-Onset (η2_p_ = 0.872) and P-Onset (η2_p_ = 0.492). After adjustment, group effects remained significant for N-Onset (η2_p_ = 0.799), P-Onset (η2_p_ = 0.513), and N-Peak (η2_p_ = 0.565). Although P-Peak and amplitude changes did not reach significance, their effect sizes (η2_p_ > 0.06 and >0.01) suggested a clinically meaningful influence. These findings support the EDX system’s sensitivity to both the presence and severity of SCI. This proof-of-concept study demonstrates the feasibility and diagnostic value of an epidural EDX platform for real-time segmental monitoring of spinal conduction. The system’s robust sensitivity to latency shifts and force-dependent modulation underscores its potential for intraoperative neuromonitoring, SCI diagnosis, and injury stratification. Its dorsal column targeting and catheter-based design also support integration into closed-loop neuromodulatory frameworks and longitudinal neurorehabilitation, providing a foundation for future clinical translation.

## Introduction 

Spinal cord injury (SCI) remains a catastrophic clinical challenge, often resulting in permanent deficits in motor, sensory, and autonomic function. Beyond the initial mechanical insult, a complex cascade of secondary pathophysiological events, including inflammation, ischemia, and excitotoxicity, drives progressive tissue damage and worsens neurological outcomes. Early management strategies prioritize mitigation of secondary injury, with optimization of spinal cord perfusion and maintenance of mean arterial pressure forming key pillars of acute neuroprotective care.^1,2^

Despite these advances, a fundamental gap persists: the absence of tools capable of providing continuous, segment-specific, real-time assessment of spinal cord functional integrity at the site of injury. Conventional modalities, such as somatosensory evoked potentials (SSEPs) and magnetic resonance imaging (MRI), provide valuable information but are inherently limited, either episodic in nature or reliant on indirect markers of neural function.^3,4^ Our group has previously developed a miniaturized near-infrared spectroscopy (NIRS) platform for monitoring spinal cord oxygenation^5^; however, while NIRS captures hemodynamic dynamics, it does not directly interrogate neural conduction.

Electrodiagnostic (EDX) approaches, particularly nerve conduction studies (NCS), are foundational for evaluating the integrity of the peripheral nervous system.^6^ These techniques quantify conduction velocity, signal amplitude, and onset latency in response to controlled electrical stimulation, enabling diagnosis of neuropathies such as demyelination, axonal injury, and conduction block.^6,7^ However, conventional EDX is restricted to peripheral pathways and cannot probe conduction within the spinal cord.

To address this unmet need, we developed a novel catheter-mounted epidural EDX sensor system for direct, segmental evaluation of spinal somatosensory conduction. This minimally invasive platform enables both stimulation and recording at targeted spinal levels, including directly over injured regions. The epidural configuration provides high signal fidelity, spatial specificity, and reduced interference from surrounding tissues, making it well-suited for intraoperative and critical care applications. Importantly, the system is sensitive to both baseline conduction and dynamic physiological or pathological perturbations.

Recent pre-clinical studies demonstrate that spinal neurophysiology can be directly interrogated at the cord surface, beyond traditional cortical SSEPs. In rats, epidural dorsal column electrically evoked compound action potentials (ECAPs) have been elicited and recorded, with thresholds and amplitudes scaling with stimulation intensity and diverging from motor thresholds, supporting sub-motor, sensory-tract activation regimes for monitoring and neuromodulation.^8,9^ Similar SSEP and motor-evoked potential (MEP) paradigms have been applied in rodent and primate SCI models, where electrophysiological signal changes correlate with injury severity and functional outcomes.^10,11^ For example, Nashmi et al. (1997) in rats and Arunkumar et al. (2001) in Macaca radiata both demonstrated that graded SCI produces predictable latency and amplitude shifts in SSEPs and MEPs corresponding to injury severity. Large-animal studies, such as the porcine spinal cord compression model by Hu et al. (2023), have successfully employed spine-to-spine evoked potentials measured above and below the lesion site, correlating with injury extent and functional outcomes.^12^ Computational modelling has further clarified how electrode geometry, contact spacing, and anatomical factors influence selective recruitment of dorsal column versus root fibers during epidural stimulation.^13^ However, no catheter-based system has yet been designed for segmental, on-injury-site stimulation and recording that enables continuous conduction monitoring at the site of injury.

In the present study, we introduce a pre-clinical proof-of-concept for this epidural EDX system in a rat model of thoracic SCI. We demonstrate its ability to capture high-fidelity electrophysiological responses across physiological, pathological (hypoxia, post-injury), and recovery conditions. These findings provide initial evidence of feasibility and diagnostic value, laying the foundation for further development and clinical translation of epidural EDX as a neurocritical care monitoring tool.

## Methods

### Development of the epidural EDX sensor system

The epidural EDX system consisted of a high-fidelity nerve conduction and electromyography (NCV/EMG) platform paired with a custom-designed epidural sensor optimized for *in vivo* spinal cord conduction monitoring. The NCV/EMG unit (NR Sign, Vancouver, Canada)^14^ is a compact, clinically validated system designed for precision neurophysiological acquisition. It supports two to four concurrent recording channels with adjustable gain and a bandwidth of 10 Hz–10 kHz. The input circuitry includes a high input impedance (>10 MΩ) and a differential amplifier with a common-mode rejection ratio exceeding 110 dB, ensuring excellent signal integrity with minimal noise. Data were digitized at sampling rates of 20–50 kHz, providing high temporal resolution suitable for capturing fine features of compound evoked potentials. The integrated stimulator delivers monophasic square-wave pulses with adjustable parameters: pulse durations from 0.05 to 1 msec, intensities up to 10 mA, and stimulation frequencies ranging from 0.5 to 50 Hz. This enabled precise and repeatable activation of spinal cord pathways across varying physiological conditions.

The custom-built epidural sensor was engineered for segmental somatosensory conduction mapping along the thoracic spinal cord in rodents. It consisted of a miniaturized, flexible catheter incorporating a copper wire electrode array configured for atraumatic epidural insertion and stable dorsal dural contact. Electrode spacing and geometry were tailored to rat spinal anatomy to optimize compound action potential (CAP) acquisition while minimizing mechanical disruption. The system enabled both epidural stimulation and real-time recording of spinal evoked responses, with sufficient fidelity to resolve waveform morphology, onset and peak latency shifts, and amplitude modulations across experimental conditions. Key conduction parameters, including onset latency, peak latency, signal amplitude, and waveform duration, were extracted from each trial to quantify functional changes in spinal cord transmission before and after injury.

### Animal model and ethical compliance

Five adult male Sprague Dawley rats (Charles River Laboratories), weighing between 450 and 515 g, were used. All animal procedures in this study were conducted in accordance with the ethical standards and regulations established by the Canadian Council on Animal Care and were approved by the University of British Columbia Animal Care Committee. Experimental protocols complied with institutional and national guidelines for the care and use of laboratory animals. All surgical interventions, perioperative monitoring, and euthanasia procedures were performed under appropriate anesthesia to minimize animal suffering. The study adhered to the principles of the 3Rs (Replacement, Reduction, Refinement) in animal research.

### Surgical preparation

Animals were induced under 4% isoflurane in 100% oxygen and maintained at 1–2% isoflurane during the experiment. Buprenorphine (0.05 mg/kg, subcutaneous) was administered pre-operatively for analgesia. The dorsal thoracic region was shaved, disinfected with 2% chlorhexidine followed by 70% ethanol, and draped using aseptic technique. Local anesthesia was achieved with a subcutaneous injection of lidocaine 2% with epinephrine (0.5 mL/kg). Corneal ointment was applied to prevent desiccation, and body temperature was maintained at 36.5–37.5°C using a rectal probe and heating pad. Heart rate and arterial oxygen saturation (SpO_2_) were continuously monitored using a pulse oximeter (MouseSTAT® Jr; Kent Scientific) attached to the hind paw.

A midline dorsal incision (∼4–5 cm) was made to expose the thoracic vertebrae. The paraspinal muscles over T8–T10 were bluntly dissected, and a T9 laminectomy was performed to expose the dura mater and underlying spinal cord.

### Experimental protocol and stimulation–recording configuration

**FIG. 1.**
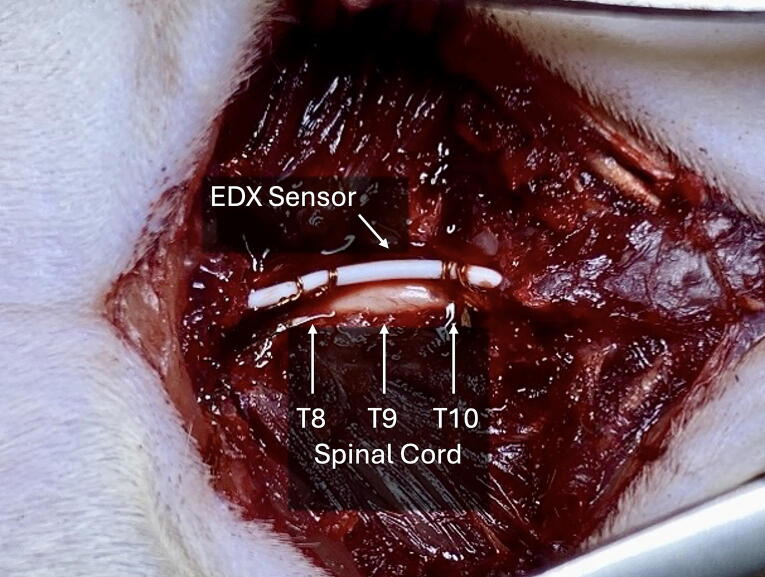
Schematic illustration of the epidural electrodiagnostic (EDX) sensor placement and experimental setup in a rat model of thoracic spinal cord injury. A miniaturized copper electrode catheter was positioned epidurally along the dorsal thoracic spinal cord, spanning the T8–T10 laminae. This configuration enabled segmental electrical stimulation and real-time recording of evoked spinal potentials at the T9 contusion site.

The EDX sensor catheter was positioned epidurally between the T8 and T10 laminae with its distal contact centered over T9 and gently opposed to the dorsal dura (Fig. 1). Electrical stimulation was delivered epidurally through this catheter (monophasic square pulses; intensity 3 mA; pulse duration within the 0.05–1 msec range supported by the stimulator), and compound evoked responses were recorded epidurally from the same catheter using a brief post-stimulus amplifier blanking window to suppress the immediate stimulus artifact, followed by standard band-pass acquisition (10 Hz–10 kHz). Stimulation was applied in 1-min trains separated by 4-min rest periods, repeated over 10-min cycles. Hypoxia (7% O_2_/93% N_2_ for 30 sec) was introduced during continuous monitoring, followed by reoxygenation and repeat stimulation cycles.

Following the pre-injury recordings, the sensor was temporarily removed, and the animal was transferred to the Infinite Horizon (IH) impactor platform (IH-0400; PSI Impactors). A moderate contusion SCI was induced at T9. The spine was stabilized using clamps at T8 and T10, and a 2.5-mm diameter stainless steel impactor tip was incrementally lowered to make contact with the dura. A standardized 4 mm height drop was applied using the IH apparatus to deliver the impact. Real-time force, velocity, and displacement parameters were recorded.

After injury, the animal was returned to the surgical platform, and the EDX sensor was reimplanted over the injury site. The stimulation protocol, including normoxic and hypoxic phases with the same electrical stimulation and recording protocol, was repeated to evaluate post-injury conduction changes. At the end of the experimental protocol, animals were euthanized via intraperitoneal injection of chloral hydrate (1 g/kg) under deep anesthesia.

### Motion control and artifact removal

Animals were maintained under a surgical plane of isoflurane anesthesia throughout the experiment. The vertebral column was rigidly stabilized with clamps at T8 and T10 during both pre- and post-injury recordings to minimize micromotion. All trials were visually monitored for trunk or limb movements at the stimulation frequency; none were observed at the 3-mA intensity used for data acquisition. The recorded waveforms consistently showed a brief, stimulus-locked near-field artifact followed by a delayed, reproducible biphasic response. Postmortem stimulation abolished the delayed component while leaving the artifact intact, confirming the biological origin of the neural response and arguing against motion contamination. Moreover, the stimulation intensity was within previously reported ranges that elicit dorsal column ECAPs below motor thresholds in rats, supporting its use as a sub-motor, sensory-tract monitoring regime.^15^

### Signal acquisition and analysis

**FIG. 2.**
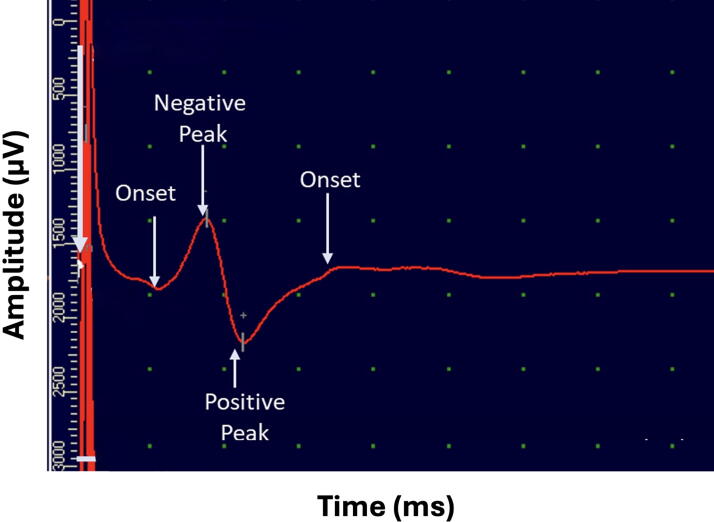
A representative epidural electrodiagnostic waveform recorded over the thoracic spinal cord. The first deflection (early component) corresponds to passive electrical conductivity through tissue, serving as a temporal reference. The second deflection (delayed component) reflects active neural conduction along somatosensory pathways. This biphasic structure enables differentiation between nonneuronal and neuronal components of the recorded signal.

Each recording exhibited two temporally distinct components. The initial component appeared immediately after stimulation and reflected passive electrical spread through surrounding tissues, serving as a reliable temporal reference. The second, consistently delayed component corresponded to the CAP generated by propagating neural activity along intact spinal pathways. This reproducible separation enabled clear discrimination of genuine neuronal responses from passive tissue conductivity, providing a robust basis for interpretation.^16^ A representative biphasic waveform illustrating these components is shown in Figure 2.

Measurement reliability was ensured by performing eight stimulation trials per condition. Intertrial variability in latency was consistently <5%, supporting high repeatability. Latency values were averaged across trials to yield stable estimates of conduction time. Electrode placement was maintained to optimize signal fidelity. Postmortem repetition of the protocol resulted in near-complete attenuation of the delayed neural component, further confirming its biological basis.

### Statistical analysis

Descriptive statistics were calculated for electrophysiological parameters (onset and peak latencies, peak-to-peak amplitude) and injury metrics across animals. For each condition, negative onset (N-Onset), negative peak (N-Peak), positive onset (P-Onset), and positive peak (P-Peak) latencies were averaged across eight trials per animal. Intertrial variability was consistently <5%, confirming recording reliability. The Shapiro–Wilk test was applied to evaluate normality of latency distributions; nonsignificant results (*p* > 0.05) indicated no evidence of deviation. Accordingly, paired *t*-tests were used to compare baseline with experimental stages across the five metrics. In addition, ANCOVA was performed to examine the effect of impact force on each metric between Baseline and Post-SCI conditions. All tests were two-tailed, with significance set at *p* < 0.05. Analyses were conducted using R Studio v2025.05.1.

## Results

SCI was induced using a standardized contusion protocol. SCI parameters were recorded in five adult rats using the IH impactor. Data include animal body weight, applied impact force, velocity at impact, and resultant displacement (Table 1). The mean impact force was 208 ± 25 kdyn, with a mean spinal cord displacement of 1128 ± 57 µm and impact velocity of 125 ± 5 mm/sec. These values confirm consistent induction of moderate thoracic contusion injury across all animals.

**Table 1.** Individual Injury Metrics

Animal	Body weight (g)	Force (kdyn)	Impact velocity (mm/sec)	Displacement (µm)
1	450	271	124	1763
2	459	208	127	1058
3	513	214	129	1199
4	456	206	117	1128
5	458	208	127	1128

Electrophysiological recordings were obtained at five distinct stages: Baseline, Post-Hypoxia, Post-SCI, Post-SCI Hypoxia, and Post-SCI Post-Hypoxia. Summary of descriptive statistics (mean and standard deviation [SD]) and Shapiro–Wilk test of normality for key EDX parameters, including negative onset latency (N-Onset), negative and positive peak latencies (N-Peak, P-Peak), positive repolarization onset (P-Onset), and peak-to-peak amplitude (Amp p–p), are presented in Table 2.

**Table 2.** Descriptive Statistics and Normality Test for Electrodiagnostic Metrics Across Stages

Latency metrics	Experimental stages	Mean	SD	Shapiro–Wilk test (*p* > 0.05)
N-Onset (msec)1	Baseline	1.17	0.10	0.166
	Post-Hypoxia	1.21	0.09	0.189
	Post-SCI	1.31	0.12	0.762
	Post-SCI Hypoxia	1.29	0.12	0.459
	Post-SCI Post-Hypoxia	1.29	0.11	0.609
N-Peak (msec)1	Baseline	1.55	0.10	0.318
	Post-Hypoxia	1.58	0.08	0.257
	Post-SCI	1.71	0.05	0.491
	Post-SCI Hypoxia	1.68	0.05	0.147
	Post-SCI Post-Hypoxia	1.68	0.05	0.427
P-Peak (msec)1	Baseline	2.21	0.24	0.186
	Post-Hypoxia	2.23	0.24	0.231
	Post-SCI	2.37	0.24	0.513
	Post-SCI Hypoxia	2.39	0.21	0.769
	Post-SCI Post-Hypoxia	2.32	0.17	0.804
P-Onset (msec)1	Baseline	3.60	0.58	0.941
	Post-Hypoxia	3.95	0.20	0.521
	Post-SCI	4.36	0.58	0.635
	Post-SCI Hypoxia	4.34	0.53	0.760
	Post-SCI Post-Hypoxia	4.33	0.56	0.592
Amp p–p 1 (µV)	Baseline	934.20	164.28	0.971
	Post-Hypoxia	907.80	162.40	0.521
	Post-SCI	881.20	209.49	0.979
	Post-SCI Hypoxia	940.40	351.17	0.692
	Post-SCI Post-Hypoxia	982.80	318.36	0.695

SCI, spinal cord injury; SD, standard deviation. 1, These latency values represent the mean of eight consecutive stimulation trials per animal.

Following SCI, significant alterations were observed in neural conduction latencies and evoked signal amplitude. The average N-Onset latency increased from 1.17 ± 0.10 msec at Baseline to 1.31 ± 0.12 msec Post-SCI (*p* = 0.015), while N-Peak latency increased from 1.55 ± 0.10 msec to 1.71 ± 0.05 msec (*p* = 0.019). Similarly, P-Onset latency was prolonged from 3.60 ± 0.58 msec to 4.36 ± 0.58 msec (*p* = 0.036), and P-Peak latency rose from 2.21 ± 0.24 msec to 2.37 ± 0.24 msec (*p* = 0.043). These latency shifts reflect delayed conduction through the injured spinal cord, likely attributable to disrupted axonal integrity and impaired saltatory propagation.

The peak-to-peak amplitude of the compound evoked response declined progressively across experimental stages, decreasing from 934.20 ± 164.28 µV at Baseline to 881.20 ± 209.49 µV Post-SCI (*p* = 0.022). Amplitude remained suppressed during the Post-SCI Hypoxia stage (940.40 ± 351.17 µV), with a modest increase observed during Post-SCI Post-Hypoxia recovery (982.80 ± 318.36 µV). No significant amplitude change was detected between Baseline and Post-Hypoxia (907.80 ± 162.40 µV, *p* = 1.000), suggesting that the most substantial electrophysiological disruption occurred in direct response to the contusion injury rather than systemic hypoxic stress.

**FIG. 3.**
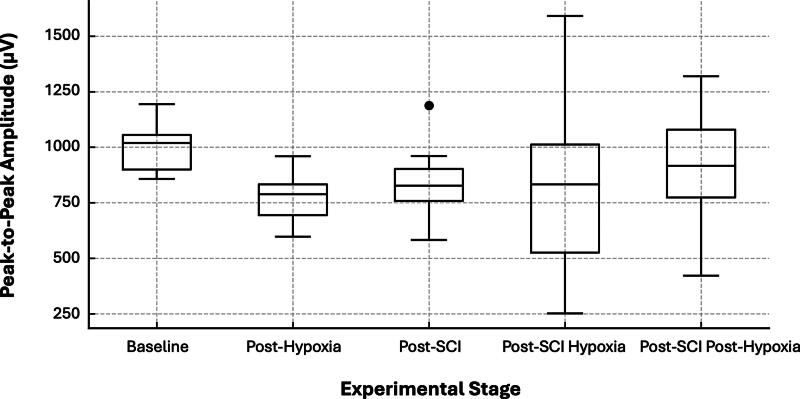
Boxplot of electrodiagnostic (EDX) signal amplitude (peak-to-peak, µV) across experimental stages. Boxplots show the distribution of peak-to-peak amplitudes of compound evoked responses recorded using the epidural EDX system in five rats (*n* = 5) across the five experimental stages: Baseline, Post-Hypoxia, Post-SCI, Post-SCI Hypoxia, and Post-SCI Post-Hypoxia. Individual animal data are overlaid as black dots. Red markers indicate the mean amplitude for each stage, with vertical bars representing ±1 standard deviation. This visualization highlights stage-specific attenuation and partial recovery of spinal conduction amplitude following contusive injury and physiological perturbations. SCI, spinal cord injury.

These findings are visually summarized in Figure 3, which shows the progression of amplitude changes across all stages using consistent y-axis scaling to enable direct comparisons. The figure illustrates the immediate suppression of signal amplitude following SCI, further reduction during post-injury hypoxia, and partial rebound in the recovery phase. Together, these patterns demonstrate both acute disruption and limited plasticity in spinal conduction following injury.

**FIG. 4.**
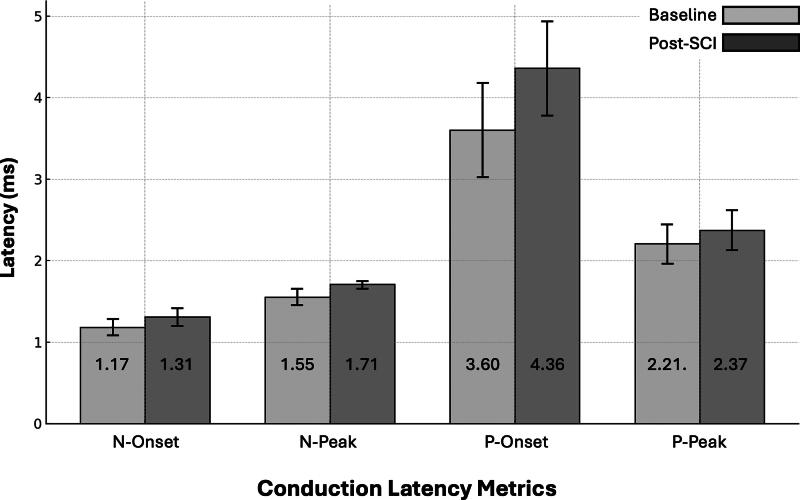
Conduction latency metrics before and after spinal cord injury (SCI). Bar plots show the mean ± standard deviation for four latency measures, N-Onset, N-Peak, P-Onset, and P-Peak, recorded at Baseline and after contusion injury (Post-SCI). SCI was associated with increased conduction latency across all measures, reflecting delayed depolarization and repolarization along the somatosensory pathway. These findings indicate impaired neural conduction consistent with injury-induced disruption of spinal signaling.

In addition to amplitude suppression, SCI induced apparent temporal shifts in waveform morphology. Both onset and peak latencies were delayed across the N and P wave components, with N-Onset latency, representing the initiation of spinal neural conduction, increasing significantly from 1.18 ± 0.01 msec at Baseline to 1.31 ± 0.11 msec Post-SCI (*p* = 0.016). This delay is indicative of impaired axonal conduction and disrupted saltatory propagation through damaged myelinated fibers. Similarly, N-Peak latency increased from 1.55 ± 0.10 msec to 1.71 ± 0.05 msec (*p* = 0.019), reinforcing its sensitivity as an electrophysiological marker of conduction slowing. Changes in the P wave components followed comparable trends: P-Onset latency rose from 3.60 ± 0.58 msec to 4.36 ± 0.58 msec (*p* = 0.038), while P-Peak latency increased from 2.21 ± 0.24 msec to 2.37 ± 0.24 msec (*p* = 0.042). These latency shifts were consistently observed across animals and confirm widespread injury-induced conduction delays in both depolarization and repolarization phases. These trends are illustrated in Figure 4, which depicts paired comparisons of both onset (A) and peak (B) latency metrics before and after SCI across the full cohort.

A paired *t-*test was conducted to compare latency metrics, N-Onset, N-Peak, P-Peak, P-Onset, and Amp, across Baseline and four experimental stages (Post-Hypoxia, Post-SCI, Post-SCI Hypoxia, Post-SCI Post-Hypoxia). Regarding N-Onset, the mean difference between Baseline and Post-SCI (*d* = 0.14, SD = 0.05) was significant (*t*^4^ = 6.08, *p* < 0.001, Cohen’s *d* = 3.04, 95% CI: [0.78, 5.27]) with a large effect (>0.80).^17^ Similarly, Post-SCI Post-Hypoxia (*d* = 0.12, SD = 0.05) and Post-SCI Hypoxia (*d* = 0.12, SD = 0.05) both differed significantly from Baseline (*t*^4^ = 5.12, *p* < 0.05, Cohen’s *d* = 2.56, 95% CI: [0.57, 4.50]; *t*^4^ = 4.90, *p* < 0.05, Cohen’s *d* = 2.45, 95% CI: [0.52, 4.33]), indicating increases of over two SDs. However, for the N-Peak metric, only the Post-SCI stage showed a significant increase from Baseline (*d* = 0.17, SD = 0.07), *t*^4^ = 5.13, *p* < 0.05, Cohen’s *d* = 2.57, 95% CI: [0.58, 4.51], reflecting a large effect. Regarding P-Peak, significant differences from Baseline were observed for Post-SCI (*d* = 0.16, SD = 0.09), *t*^4^ = 4.06, *p* < 0.05, Cohen’s *d* = 2.03, 95% CI: [0.33, 3.67]) and Post-SCI Post-Hypoxia (*d* = 0.11, SD = 0.07), *t*^4^ = 3.39, *p* < 0.05, Cohen’s *d* = 1.70, 95% CI: [0.16, 3.15], both having strong effects. Other comparisons across latency metrics were nonsignificant (*p* > 0.05), still presented small to moderate effects (Cohen’s *d* >0.2), except for Baseline versus Post-SCI Hypoxia for Amp p–p1 (µV), demonstrating a negligible effect (*d* = −0.05) (Cohen’s *d* between −0.15 and +0.15 is considered a negligible effect).^17^ According to widely accepted thresholds of Cohen’s *d* effect size (*d* ≈ 0.2 small, *d* ≈ 0.5 medium, *d* ≈ 0.8 large), most observed effects remained meaningful. These results suggest that increasing sample size and statistical power would enable more precise detection of meaningful differences. Table 3 summarizes the paired-samples *t*-tests.

**Table 3.** Paired Samples *T*-Test of Electrodiagnostic Parameters

Latency metrics	Experimental stages Baseline vs.	Mean differences	SD	t	*p* Value	Cohen’s d	95% confidence interval
							Lower, Upper
N-Onset (msec)	Post-Hypoxia	0.04	0.04	2.13	0.10	1.07	[−0.186, 2.237]
	Post-SCI	0.14	0.05	6.08^*^	0.00	3.04	[0.782, 5.268]
	Post-SCI Hypoxia	0.12	0.05	4.90^*^	0.01	2.45	[0.524, 4.326]
	Post-SCI Post-Hypoxia	0.12	0.05	5.12^*^	0.01	2.56	[0.574, 4.500]
N-Peak (msec)	Post-Hypoxia	0.03	0.04	1.75	0.15	0.88	[−0.304, 1.979]
	Post-SCI	0.17	0.07	5.13^*^	0.01	2.57	[0.576, 4.508]
	Post-SCI Hypoxia	0.13	0.12	2.39	0.08	1.20	[−0.109, 2.419]
	Post-SCI Post-Hypoxia	0.13	0.12	2.39	0.08	1.20	[−0.109, 2.419]
P-Peak (msec)	Post-Hypoxia	0.02	0.03	1.76	0.15	0.88	[−0.300, 1.986]
	Post-SCI	0.16	0.09	4.06^*^	0.02	2.03	[0.329, 3.667]
	Post-SCI Hypoxia	0.18	0.18	2.27	0.09	1.14	[−0.144, 2.334]
	Post-SCI Post-Hypoxia	0.11	0.07	3.39^*^	0.03	1.70	[0.163, 3.154]
P-Onset (msec)	Post-Hypoxia	0.36	0.51	1.58	0.19	0.79	[−0.359, 1.868]
	Post-SCI	0.76	0.67	2.53	0.06	1.27	[−0.069, 2.519]
	Post-SCI Hypoxia	0.75	0.64	2.61	0.06	1.31	[−0.046, 2.576]
	Post-SCI Post-Hypoxia	0.73	0.64	2.54	0.06	1.27	[−0.066, 2.526]
Amp p–p (µV)	Post-Hypoxia	5.19	20.12	1.29	0.21	0.65	[−0.458, 1.684]
	Post-SCI	10.35	73.74	0.70	0.49	0.35	[−0.677, 1.338]
	Post-SCI Hypoxia	−1.48	84.24	−0.09	0.93	−0.05	[−1.023, 0.938]
	Post-SCI Post-Hypoxia	−9.94	86.62	−0.57	0.57	−0.29	[−1.267, 0.730]

^*^ *p* < 0.05.

SCI, spinal cord injury; SD, standard deviation.

**FIG. 5.**
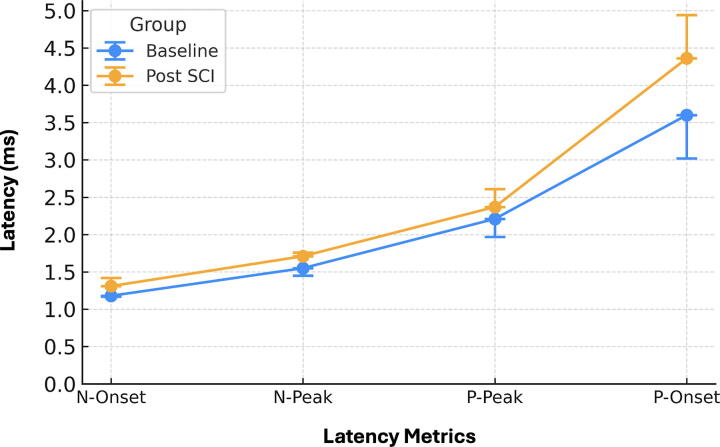
Post-SCI changes in electrodiagnostic signal parameters compared to Baseline. Parameters include latencies of negative onset (N-Onset), negative peak (N-Peak), positive peak (P-Peak), and positive onset (P-Onset). Data are shown as mean ± standard deviation (*n* = 5). SCI, spinal cord injury.

Figure 5 illustrates that Post-SCI values are consistently higher than Baseline across all metrics and steadily increase from N-Onset to P-Onset. While N-Onset and N-Peak show slight increases, Post-SCI values become more pronounced at P-Peak, and for P-Onset, the Post-SCI values increase notably above Baseline.

**FIG 6.**
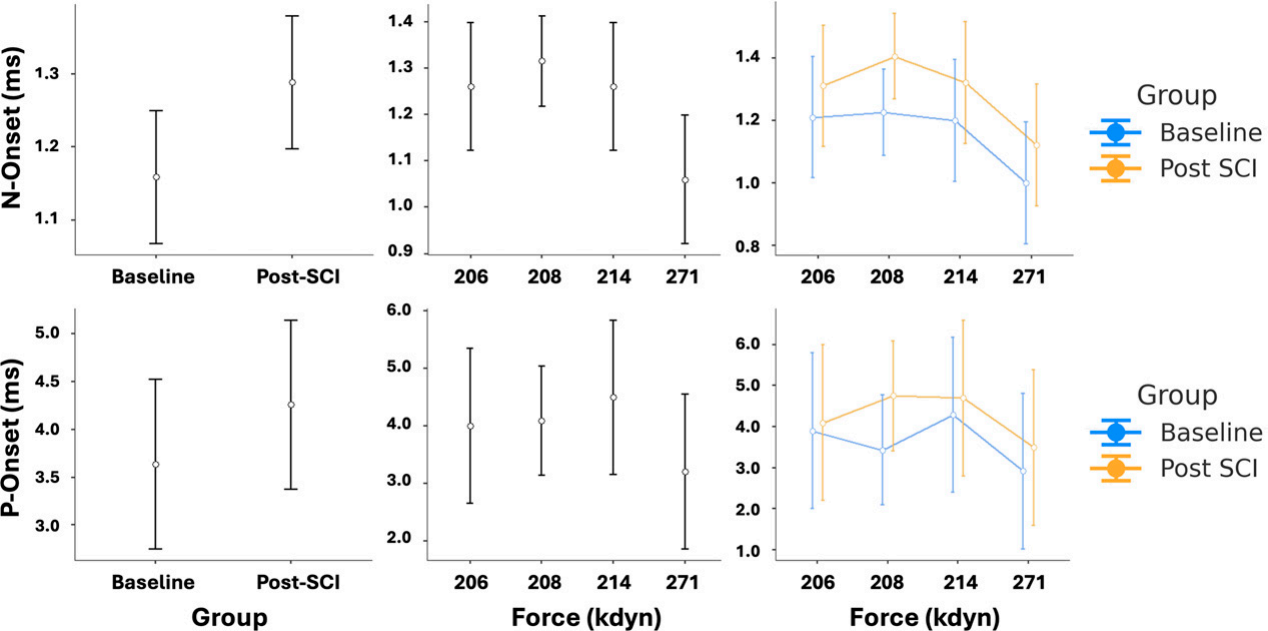
Influence of injury force on N-Onset and P-Onset latencies before and after spinal cord injury (SCI). Error bar plots depict latency values for N-Onset (top row) and P-Onset (bottom row) metrics, comparing Baseline and Post-SCI stages across animals. Left column: Mean latency values (± standard deviation) at Baseline and Post-SCI stages, aggregated across all animals (*n* = 5). Middle column: Latency values plotted against delivered contusion force (kdyn), illustrating inter-animal variability. Right column: Group-wise comparison of latency values across injury forces. These plots support the results of the ANCOVA analysis, indicating that contusion force had a significant effect on both N-Onset and P-Onset latencies, with a significant group effect (Baseline vs. Post-SCI) remaining after controlling for force. ANCOVA, analysis of covariance.

Further analysis using one-way analysis of covariance (ANCOVA) was conducted to compare Post-SCI and Baseline values for each of the five electrophysiological metrics while controlling for force as a covariate. The analysis revealed that force exerted a statistically significant effect on both N-Onset, *F*(1, 7) = 47.5, *p* < 0.001, η2_p_ = 0.872, and P-Onset, *F*(1, 7) = 6.78, *p* < 0.05, η2_p_ = 0.492, each representing a large effect size (η2_p_ > 0.14).^17^ After controlling for force, the group effect (Baseline vs. Post-SCI) remained statistically significant for both N-Onset, *F*(1, 7) = 27.9, *p* < 0.01, η2_p_ = 0.799, and P-Onset, *F*(1, 7) = 7.37, *p* < 0.05, η2_p_ = 0.513, again indicating large effects (Fig. 6). For the N-Peak metric, a significant group effect was also observed, *F*(1, 7) = 9.11, *p* < 0.05, η2_p_ = 0.565, while force had no significant effect (*p* > 0.05, η2_p_ < 0.01). Although the group and force effects were not statistically significant for the P-Peak metric (*p* > 0.05), the observed effect sizes (η2_p_ = 0.132 and 0.076, respectively) exceeded the minimum threshold (η2_p_ > 0.06), suggesting potentially meaningful, albeit nonsignificant, influences. Similarly, for amplitude (peak-to-peak), both force (η2_p_ = 0.037) and group (η2_p_ = 0.025) yielded minor but nonnegligible effects (η2_p_ > 0.01), even though they did not reach statistical significance.

Together, these findings suggest that force may serve as a significant covariate in explaining Post-SCI changes in certain conduction parameters, especially N-Onset and P-Onset latency. The modest yet nontrivial effect sizes observed for other metrics imply that with a larger sample size and enhanced statistical power, additional significant relationships may be uncovered.

## Discussion

EDX modalities such as electroencephalography, EMG, and NCV testing are foundational tools in neuromuscular and clinical neurophysiology. Among these, needle EMG and NCV studies are considered diagnostic gold standards due to their high sensitivity and specificity for detecting disorders of peripheral nerves and motor units. These techniques offer excellent temporal and spatial resolution for identifying conduction abnormalities, demyelination, and axonal injury.^18^ However, conventional EDX approaches are limited to peripheral assessments and cannot capture segmental conduction changes within the spinal cord or central neural structures.

To address this limitation, we developed a novel catheter-based epidural EDX system for continuous, segment-specific monitoring of spinal somatosensory conduction. Evaluated in a pre-clinical rat model of thoracic SCI, the system consistently recorded high-fidelity compound spinal evoked potentials across multiple physiological and pathological conditions. Unlike SSEPs or MRI, which lack the temporal and spatial resolution for dynamic, localized conduction monitoring, our EDX platform provides direct, high-fidelity insight into spinal segment-level conduction abnormalities.^19^

Following SCI, consistent and statistically significant increases in conduction latencies were observed. N-Onset latency increased from 1.18 ± 0.01 msec at Baseline to 1.31 ± 0.11 msec post-injury (*p* = 0.016), while N-Peak latency rose from 1.55 ± 0.10 msec to 1.71 ± 0.05 msec (*p* = 0.019), reflecting conduction slowing consistent with axonal damage and myelin disruption, hallmarks of traumatic SCI.^20^ Similarly, P-Peak latency increased from 2.21 ± 0.24 msec to 2.37 ± 0.24 msec (*p* = 0.042), and P-Onset latency rose from 3.60 ± 0.58 msec to 4.36 ± 0.58 msec (*p* = 0.038). These changes indicate that both depolarization-related (N-Onset, N-Peak) and repolarization-related (P-Peak, P-Onset) components of the compound signal were impacted by SCI, with particularly robust delays in N-Onset and P-Onset. The differential sensitivity of these components supports the interpretation that active neural mechanisms, such as sodium channel activation and membrane depolarization, may be more susceptible to injury than passive, glial-mediated repolarization processes.^21^

Amplitude variability was observed across experimental stages, with a statistically significant decrease in compound evoked response peak-to-peak amplitude from 934.20 ± 164.28 µV at Baseline to 881.20 ± 209.49 µV Post-SCI (*p* = 0.022). This reduction reflects an acute disruption of synchronous neural transmission, consistent with impaired axonal conduction and reduced recruitment of functional fibers following contusion injury. Amplitude remained suppressed during the Post-SCI Hypoxia phase (940.40 ± 351.17 µV), and while a modest increase was observed during the Post-SCI Post-Hypoxia recovery phase (982.80 ± 318.36 µV), values did not fully return to baseline. Importantly, no significant difference in amplitude was found between Baseline and Post-Hypoxia (907.80 ± 162.40 µV, *p* = 1.000), suggesting that the principal electrophysiological disruption was attributable to mechanical trauma rather than a short transient systemic hypoxia. These findings support the sensitivity of peak-to-peak amplitude as an indicator of injury-induced conduction failure, and they highlight the relative stability of the EDX system under nontraumatic physiological stressors. Amplitude suppression aligns with the well-documented pathophysiology of SCI, where primary mechanical trauma is followed by secondary cascades, including ischemia, inflammation, excitotoxicity, and oxidative stress, that contribute to further neuronal dysfunction and tissue damage.^21–23^

Furthermore, ANCOVA results revealed that injury force significantly affected both N-Onset and P-Onset latencies, indicating that the severity of mechanical impact modulates post-injury conduction delays. The effect of force was especially pronounced in N-Onset latency, *F*(1, 7) = 47.5, *p* < 0.001, η2_p_ = 0.872, and also significant for P-Onset, *F*(1, 7) = 6.78, *p* < 0.05, η2_p_ = 0.492, indicating large effect sizes. These findings underscore the utility of the EDX platform not only for detecting the presence of SCI but also for quantifying the severity of injury, consistent with prior studies demonstrating a dose-dependent relationship between impact force and conduction delay in SCI models.^16^

These electrophysiological alterations align with known mechanisms of acute SCI. The observed increases in onset and peak latencies reflect a pathological slowing of neural conduction attributable to axonal disruption, demyelination, and conduction block within affected spinal pathways. Mechanical trauma to the spinal cord causes immediate structural damage to axonal membranes and voltage-gated ion channels, particularly within the dorsal columns populated by large-diameter, myelinated fibers. This impairs saltatory conduction and prolongs the time required for action potential propagation, manifesting as delayed N-Onset and N-Peak latencies in epidurally recorded evoked responses.^20,21^ Reductions in signal amplitude are likely attributable to both a loss of viable conducting axons and temporal desynchronization of residual fibers due to heterogeneous damage across the spinal segment.^22,23^ Secondary injury cascades, characterized by ischemia, excitotoxicity, inflammatory cytokine release, and ionic imbalances, further disrupt axonal conduction and compromise myelin integrity.^21,24,25^ Amplitude attenuation may also reflect reduced recruitment of interneuronal and descending pathways following axonal transection and synaptic disconnection, which diminishes the spatial summation of evoked activity at the epidural surface. These patterns of latency delay and amplitude suppression are consistent with prior electrophysiological studies in animal models of SCI and correlate with histopathological indicators of white matter injury and impaired functional recovery.^26,27^

Potential contamination by stimulation-evoked movements is an important consideration for epidural recordings. In our preparation, deep anesthesia, rigid vertebral stabilization, consistent biphasic response morphology after the stimulus artifact, and loss of the delayed component postmortem together argue against motion as the source of the recorded conduction signals. Moreover, published rat studies show that epidural dorsal column ECAPs can be recorded at currents below motor thresholds, consistent with our stimulation regime.^15^

Together, these results demonstrate the feasibility of using epidural EDX signals as sensitive, segment-level biomarkers of spinal conduction failure. The system’s ability to provide continuous, localized, and minimally invasive monitoring positions it as a valuable complement to existing tools such as SSEPs and MRI. Unlike cortical evoked responses, which are susceptible to anesthesia effects and provide only indirect insights into spinal conduction, this EDX approach enables dynamic, high-resolution characterization of spinal electrophysiology, offering a promising avenue for both pre-clinical research and future clinical translation.

### Limitations and mitigation strategies

While promising, this study has limitations. The small sample size (*n* = 5) limits statistical power. Larger cohorts are needed to evaluate interindividual variability, stratify injury severities, and define diagnostic thresholds. The use of an acute, nonsurvival protocol restricted our ability to assess long-term changes such as neuroplasticity or secondary degeneration. Future studies should incorporate chronic survival models with longitudinal measurements. The absence of histological or imaging validation limits the correlation between electrophysiological findings and underlying structural changes. The integration of histological analysis (e.g., myelin density, axonal integrity) with imaging techniques, such as diffusion tensor imaging (DTI), will be essential to establish structure–function relationships. Although electrode placement was standardized, variability in depth and contact pressure may have contributed to signal fluctuations. These challenges could be mitigated using image-guided placement, impedance feedback, or anatomically conformable arrays. The brief hypoxic challenge (30 sec at 7% FiO_2_) may have been insufficient to elicit robust physiological responses; future studies should apply extended or graded hypoxic paradigms, potentially coupled with perfusion monitoring. The 20 kHz sampling rate provided reliable waveform resolution, but higher acquisition frequencies (e.g., ≥500 kHz) may reveal subtler features of conduction dynamics. Stimulus artifact contamination, likely due to the proximity of stimulation and recording sites, could be addressed by increasing spatial separation, employing bipolar stimulation, or incorporating higher-bandwidth amplifiers. Although we did not record concurrent EMG or accelerometry in this feasibility study, future iterations will incorporate time-locked EMG from distal hindlimb muscles and/or table/vertebral accelerometry to rigorously exclude motion artifacts and to empirically define sub-motor stimulation windows in each animal.^15^ Finally, mechanical enhancements, such as flexible, adhesive-backed electrodes and modular connectors, will improve sensor stability and clinical usability.

### Translational potential and expanded applications

The dorsal placement of the epidural catheter array enables targeted access to the dorsal columns, white matter tracts critical for proprioception, vibration, and fine touch. This anatomical orientation enables the monitoring of ascending somatosensory signals with high fidelity, potentially extending the utility of this system to conditions affecting posterior cord conduction.^28^ Real-time dorsal column monitoring could be valuable in surgical or intensive care contexts where posterior cord perfusion is at risk, as well as in chronic neurodegenerative disorders where demyelination or axonal degeneration of the dorsal columns is a hallmark of the disease.^29^ Thus, beyond trauma, this platform may offer a versatile tool for diagnosing dorsal cord injuries, ensuring intraoperative safety, and facilitating longitudinal functional assessment.

**FIG. 7.**
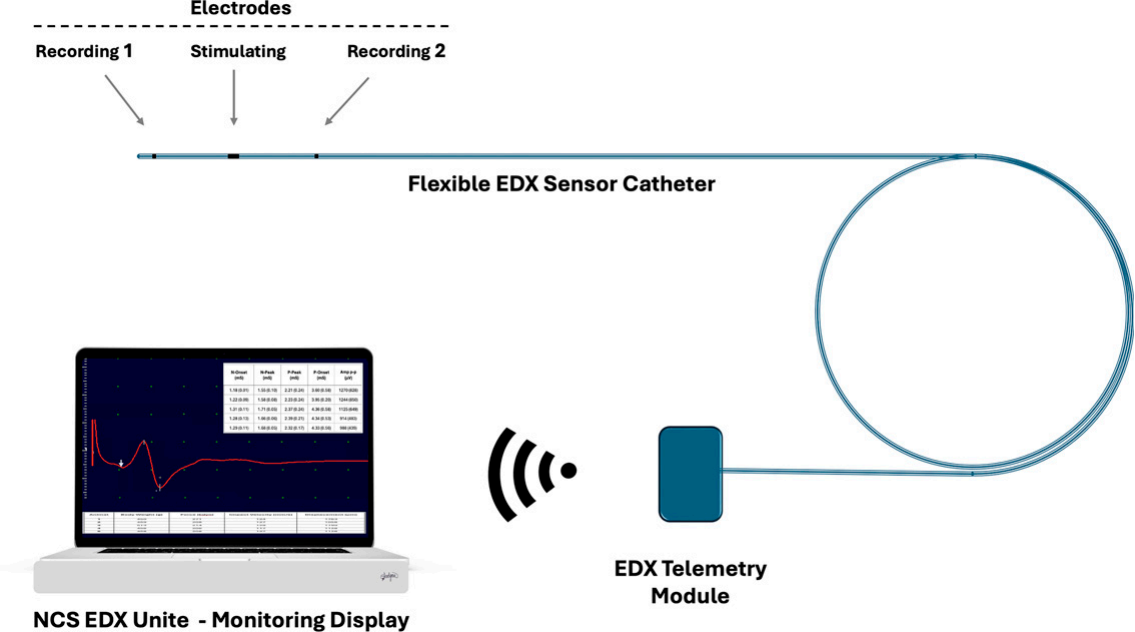
Schematic of the epidural EDX system for spinal cord monitoring. The system consists of a flexible EDX sensor catheter containing three epidural electrodes: two for recording compound spinal evoked potentials and one for stimulation, used for segmental spinal cord neural conduction assessment at two adjacent sites. The catheter interfaces with an EDX telemetry module, which wirelessly transmits recorded neural signals to the NCS EDX Unit for real-time waveform display, signal quantification, and clinical interpretation. EDX, electrodiagnostic; NCS, nerve conduction studies.

From a clinical perspective, this EDX system may fulfill a critical unmet need in multiple domains. Figure 7 illustrates an EDX system sensor setup, comprising a flexible sensor catheter, a wireless telemetric module, and an EDX hardware and computer unit.

In acute SCI management, the system can be deployed intraoperatively or in the intensive care unit to monitor evolving conduction deficits at the site of injury, guiding surgical decompression or pharmacological intervention in real time. Intraoperative applications could extend to spinal deformity correction, tumor resection, or vascular procedures with a high risk of spinal ischemia, scenarios in which segmental, real-time monitoring of conduction through specific spinal levels is currently unattainable.^30^ For patients with indwelling instrumentation, the ability to detect delayed conduction compromise due to implant migration, scar formation, or adjacent segment disease could inform early reintervention strategies. In experimental and outpatient settings, the system could enable longitudinal tracking of electrophysiological recovery during physical therapy or interventions such as stem cell transplantation, remyelination therapies, or neurotrophic delivery. Such objective measurements, including the reappearance or changes in evoked potential latencies and amplitudes, have been documented in pre-clinical models receiving mesenchymal stem cell therapy, where transcranial MEPs recovered earlier in treated rats compared to controls over 28 days.^27^ Moreover, expert reviews emphasize the increasing importance of electrophysiological tools as biomarkers in SCI clinical trials, particularly for patient stratification, monitoring adverse events, and assessing therapeutic efficacy in regenerative and remyelination studies.^31^

Meanwhile, integrating this EDX platform with epidural electrical stimulation (EES) technologies offers a unique opportunity to develop a closed-loop neuromodulatory interface. EES has shown therapeutic potential in restoring motor and autonomic function after SCI; however, current implementations are typically open-loop and lack physiological feedback.^32^ By pairing EES with this sensor’s electrophysiological readouts, clinicians could dynamically modulate stimulation parameters in real time based on conduction status, enhancing efficacy while reducing adverse effects. Furthermore, this system could detect early indicators of neural fatigue, delayed conduction, or emerging secondary injuries, enabling anticipatory therapeutic adjustments. Such bidirectional interfaces could ultimately transform spinal cord devices from static stimulators into responsive, smart neurotherapeutic systems.

In the long term, this system could evolve into a closed-loop interface for neuromodulation, wherein electrophysiological signals would not only be monitored but also used to trigger therapeutic interventions, transforming the device from a diagnostic sensor into an active component of neurorestorative therapy. This vision highlights the broader objective of our work: to develop an intelligent, implantable neural interface capable of delivering real-time, objective assessments of spinal cord conduction, with the potential to transform clinical management and treatment approaches for spinal cord and central nervous system disorders.

### Future directions

Further development of this epidural EDX platform will require evaluation under broader and more clinically relevant conditions. These include longitudinal survival models to assess chronic injury progression and recovery, variable injury severities to define diagnostic thresholds, and additional physiological perturbations such as inflammation, ischemia, and pharmacological modulation. Comparative studies against conventional neurophysiological modalities, such as SSEPs, spinal MRI, or DTI, will be essential to benchmark the system’s diagnostic sensitivity, spatial resolution, and clinical utility. Future iterations should explore wireless data transmission, miniaturization for long-term implantation, and integration with multimodal physiological sensors.

**FIG. 8.**
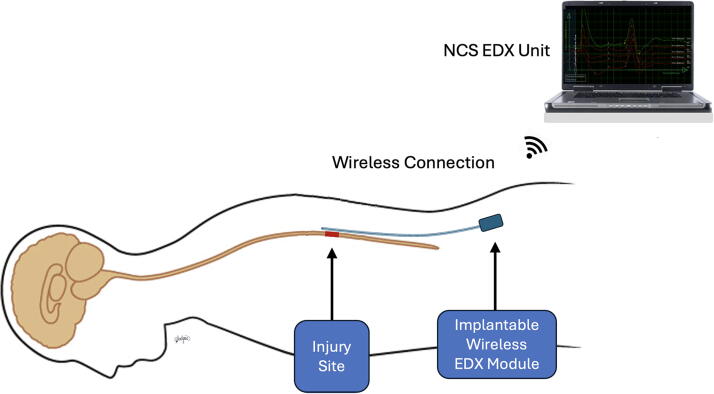
Hypothetical design of an implantable epidural EDX system for spinal cord monitoring. The system features an epidural sensor that records spinal conduction signals and transmits data wirelessly to an external computing interface for real-time analysis and clinical decision-making. EDX, electrodiagnostic.

Figure 8 illustrates the long-term translational vision of this technology: a fully implantable EDX system for continuous, closed-loop monitoring of spinal cord conduction. This envisioned system would consist of a dorsal epidural array interfaced with a subcutaneous telemetry module, wirelessly transmitting conduction metrics to external displays or neuromodulatory controllers. Such a device would enable real-time, segmentally localized assessment of spinal cord function in patients with acute or chronic SCI, as well as in other central nervous system disorders such as multiple sclerosis or transverse myelitis. By providing dynamic electrophysiological data, the system could serve as a functional biomarker for injury severity, treatment response, and neurorehabilitation progress.

## Conclusion

This study demonstrates the feasibility and diagnostic utility of a novel catheter-based epidural EDX system for real-time, segmental monitoring of spinal cord conduction *in vivo*. In a pre-clinical rat model of acute thoracic SCI, the EDX platform reliably recorded high-fidelity compound evoked potentials across baseline, hypoxic, and contusive states. SCI induced significant and sustained delays in both depolarization-related (N-Onset, N-Peak) and repolarization-related (P-Onset, P-Peak) latency parameters, with especially pronounced changes in N-Onset and P-Onset. These latency shifts, coupled with transient reductions in signal amplitude, reflect impaired axonal conduction and partial demyelination consistent with known pathophysiological mechanisms of SCI. Amplitude suppression was most pronounced immediately after injury, while latency prolongation persisted across post-injury phases. ANCOVA analyses further revealed that the severity of mechanical insult, quantified by contusion force, significantly modulated both N-Onset and P-Onset latency values, highlighting the system’s capacity to differentiate injury severity.

Critically, the epidural placement of the EDX catheter over the dorsal columns allows for segment-specific tracking of ascending spinal conduction, distinguishing this system from conventional cortical or peripheral neurophysiological monitoring tools. This design feature improves its suitability for intraoperative neuromonitoring, acute neurotrauma assessment, and real-time continuous evaluation of spinal cord function in both experimental and clinical settings. Furthermore, the system’s compact, catheter-based architecture supports its future integration into closed-loop neuromodulation platforms, potentially enabling transition from passive monitoring to real-time therapeutic intervention.

Together, these findings confirm that the EDX system is a scalable and translationally feasible technology for enhancing precision diagnostics and neurorestorative approaches in spinal cord diseases. Future studies should extend their application to chronic injury models, correlate electrophysiological changes with histopathology and behavioral outcomes, and evaluate their responsiveness to therapeutic modulation. Ultimately, this platform offers a promising avenue for improving diagnostic resolution, prognostic accuracy, and therapeutic guidance in the management of SCI and other central nervous system pathologies.

## Abbreviations Used

ANCOVA: Analysis of covariance
CAP: Compound action potential
ECAP: Electrically evoked compound action potential
EDX: Electrodiagnostic
EES: Epidural electrical stimulation
EMG: Electromyography
FiO_2_: Fraction of inspired oxygen
IH: Infinite Horizon (impactor device)
MRI: Magnetic resonance imaging
NCV: Nerve conduction velocity
NIRS: Near-infrared spectroscopy
p–p: Peak-to-peak
SCI: Spinal cord injury
SpO_2_: Peripheral oxygen saturation
SSEP: Somatosensory evoked potential
tDCS: Transcranial direct current stimulation
